# Research investigating individual device preference and e-learning quality perception: can a one-solution-fits-all e-learning solution work?

**DOI:** 10.1016/j.heliyon.2021.e07343

**Published:** 2021-06-18

**Authors:** Samnan Ali, Stephen R. Gulliver, M. Amaad Uppal, Muhammad Basir

**Affiliations:** a GC University, Lahore, Pakistan; b University of Reading, Reading, UK

**Keywords:** Face-to-Face learning, E-learning, Culture value dimensions, HE services quality indicators, Quality perception, Quality indicators

## Abstract

**Background:**

COVID-19 caused a paradigm shift for educators, and raised many questions about the future of technology in the delivery of educational content. Literature highlights numerous benefits of using e-learning solutions, yet many still consider ‘online learning’ as the cheap/‘low-quality’ alternative to traditional ‘face-to-face’ models. In this research we ask two questions that are critical to the effective development of future e-learning solutions: Do students prefer face-to-face (traditional) learning methods or e-learning technology enabled solutions? Does perception of e-learning, and/or device preference, vary between individuals?

**Methods:**

A three part, quantitative questionnaire was developed, based on previously used questionnaire items, which collected: demographic data, student preference concerning learning, and individual variance - via use of the Cultural Value (CV) Scale dimension test. Data was collected from 518 participants using convenience sampling from a range of universities in Pakistan. EFA and CFA showed that questions and factor loading was good. CV Scale results show clear loading and model fit at the individual level, allowing application of results beyond Pakistan.

**Results:**

By considering the CV Scale dimensions, our results highlighted three distinct technology preference clusters: i) students, with a high-power distance score, who prefer traditional face-to-face teaching methods; ii) students with low power distance and high uncertainty avoidance scores, who prefer use of e-learning on their mobile devices, and iii) students with low power distance and low uncertainty avoidance scored, who prefer to use laptop devices.

**Conclusions:**

This paper highlights that the majority of students are happy to engage with online blended learning solutions, however a one-solution fits all approach to technology use in education fail to satisfy the interaction preferences need of all student groups. Only by embracing flexible and mixed blend delivery models, supporting interaction across a range of pervasive devices, can we maximize student perception towards education service provision.

## 1 Introduction

Education is crucial for development, at both individual and national levels [1, 2]. Accordingly, education is significantly important to equip any population with the skills they need for life [3, 4]. To support dissemination of knowledge to all, especially within sparsely populated and/or developing countries, education institutions have increasingly focused on development of e-learning solutions [5]. E-learning can support learners in remote locations, learners with unpredictable or unsociable working hours, and those with ill health and/or in isolation. E-learning practically offers flexible through-life education solutions [6] by delivering material remotely [7], and interaction is only constrained by the technology used to facilitate learning [8].

Although the number of people signing up for E-learning courses has increased exponentially [9], numerous researchers have highlighted concerns as to the effectiveness of e-learning solutions [10]. Studies highlight concern that remote e-learning students feel secluded [11, 12], and often suffer in their studies due to the low levels of student-teacher interactivity [13]. The student drop-out rate for e-learning courses is 10–20% higher than that of traditional face-to-face courses [14, 15] - a trend amplified to nearly 95% for students undertaking Massive Open Online Courses (MOOC) courses [16]. As a result, some consider e-learning models as the ‘cheap’, low-cost, low-quality alternative to traditional ‘face-to-face’ education [17, 18, 19].

Literature shows that student retention is highly correlated with student perception of quality [20], which means that education providers can only attract and retain online students if they provide educational services that are perceived to reliably meet student requirements and add value to students. Since interaction capability and user quality perception is dependent on the device used to access the service [21], the following research questions must be considered: Do students prefer face-to-face (traditional) learning methods or e-learning technology enabled solutions? and Does perception of e-learning, and/or device preference, vary between individuals?

Since there is very limited literature relating to the impact of individual difference on device preference, it is extremely difficult to form a clear hypothesis based on existing theory. Accordingly, the authors aim to investigate whether individual's device preference, and education quality perception, was impacted by individual level cultural difference. Numerous education quality indicators have been described in literature for use to assess the quality of education provided by higher education institutions (HEIs). Fricke [22] proposed use of four components when defining quality, i.e. Learner, Topic, Learning environment, and Student Goal; highlighting how environmental factors must be considered. Jung [23] identify seven quality dimensions, Institutional support, Course development, Course structure, Teaching and learning, Student support, Faculty support, Evaluation & assessment. Uppal et al. [24] extended the SERVQUAL model to assess the e-learning quality by adding two factors Learning Content and Course Website. Hadullo et al. [25] categorized: Course Design, Content support, social support, and Student Characteristics, Instructor Characteristics, Technician Characteristics, Course Assessment and Institutional factors that influence the e-learning quality. In the context of e-learning Ehlers [26] defined seven quality concept fields, i.e., Tutor Support, Cooperation, Technology, Costs, Information Transparency, Course Structure, and Didactics. Although Ehler's concepts cover a wide range of e-learning factors, assessment does not actually consider the student's perception; instead considering quality from the education provider perspective. To the best of our knowledge, the only set of education quality indicators that considered higher education service quality from the student's perspective was proposed by Kwan and Ng [27]; subsequently developed by Watson, Saldaña, & Harvey [28] and validated by Tan & Kek [29]. Kwan and Ng [27] proposed that, to assess student's perspective of education quality we must consider: course content (material in course, module components offered, etc.); facilities (i.e. library, recreational facilities, sports, etc.); Lecturer concern for students (i.e. personal attachment towards students, talking with students after class, etc.); social activities (i.e. interactions with fellow students through events, clubs and societies, etc.); communication with university (i.e. student's communication with University management, etc.); assessment (i.e. exam/quizzes/assignments/feedback, etc.); counselling services (i.e. the range of help provided by advisor, etc.); instruction medium (i.e. language and channels used in instruction of education, etc.); and people (i.e. meeting people with similar interests, making close friends, etc.). Numerous studies have used Kwan and Ng's quality indicators to assess, from the student's perspective, different aspects of higher education delivered by HEIs [24, 30, 31, 32]. Moreover, the Kwan and Ng quality indicator has been shown to work in 3 different national cultural setting (i.e., US, China, and Hong Kong), which supports its international application and use. To the best of our knowledge, however, Kwan and Ng's [27] indicators have never been applied to the assessment of online based e-learning solutions. In this study we aim to apply and adapt Kwan and Ng's higher education service (HES) quality indicators in order to assess student perception of face-to-face and e-learning solution quality, across a range of e-learning devices, i.e. in order to answer the stated research questions.

## 2 Methods

### 2.1 Instrument and sample design

A questionnaire was developed, which consisted of three parts. The first part related to categorical collection of general demographic data, e.g. Age, Gender, Education level, etc. The second part (see appendix A) measured the student's preference concerning i) use of traditional face to face teaching, and ii) devices (including consideration of Television, Radio, Desktop, Laptop, Tablet, and Mobile), for delivery of the Higher Educational Services defined by Kwan and Ng [27] - i.e. Facilities, Lecturer Concern for Students, Social Activities, Communication with University, Assessment, Counselling Services, and People. Data concerning ‘Instruction Medium’, which was the final Kwan and Ng category, was not captured since instruction medium is assessed by capturing feedback concerning student learning mode and e-learning device preference. Questions were adapted from Kwan and Ng (1999) for use with e-learning, and data was captured using a 5-point Likert scale, with 1 and 5 respectively representing strongly disagree and strongly agree. The third part of the questionnaire was used to investigate the impact of individual variance. Individual culture is essential to information assimilation within the human semiosis processes [33]. Since not everyone from a specific country, or institution has the same background, feelings, biases, beliefs, and/or thoughts, assessment at the individual level is critical. Unfortunately, most cultural questionnaires are designed for use at the macro-level, and as such do not allow individual (micro) level analysis [34]. To address this problem, Yoo et al. [35] developed the Cultural Value (CV) scale questionnaire (see Appendix B), which categorizes the individual using the five main national culture dimensions used by Hofstede; i.e., Power distance (PD), Masculinity v Femininity (MF), Individualism vs Collectivism (IC), Uncertainty Avoidance (UA), Long term vs Short-term orientation (LS) – (see [36]). Consideration of CV dimensions helps the researchers consider individual level variation, and facilities consideration of whether difference exists in e-learning device preference, and/or user quality perception. In this study the unedited CV scale questionnaire (see appendix B) was used to measure individual difference.

Fowler [37] suggests that four factors, i.e., sampling choice, sample frame, sample size and response rate, should be carefully considered when selecting respondents for any study. This study applies non-probabilistic convenience sampling. Although a large sample size does not ensure precision, use of a small sample size results in a greater chance of failure and/or incorrect interpretation of findings. Sample size was initially calculated using the “Raosoft sample size calculator”; as used in [38, 39]. The Raosoft sample size calculator calculates the sample size based on the margin of error, confidence level, population, and the response distribution (Fatoki & Chindoga, 2011). Using a margin of error at 5%, a confidence level of 95%, and a total population of 1.3 million - the recommended sample size was 385. Hair et al. [40] state that if the number of SEM constructs is more than 6, the size of the sample should be at least 400. As such, and to allow for capture error, we targeted a sample size of above 500.

### 2.2 Demographics

In practice data was collected from 560 business students. 42 responses were discarded due to data issues, including skewness, normality, and missing values. Accordingly, data from 518 participants was analyzed. The largest group of students were aged 18–25 (69.3%), which aligns well with the age of students in higher education. 23.6% was between 15-20, 69.3% of students were between 21-25, and 4.4% of students were aged 26–30. The lowest category was aged 31 and over. 67.4% of students were enrolled in the Bachelors (BBA) programme, and 22.4% were enrolled in the Master (MBA) programme. The rest (10.3%) of respondents were enrolled in Professional degree programmes, i.e., Executive MBA and MBA Engineering. All participants had previously had exposure to both face-to-face and online e-learning delivery of content as part of their university course.

### 2.3 Validating use of CV scale

To justify the use of the CV scale, and validate the question constructs (see Appendix B), Exploratory Factor Analysis (EFA) and Confirmatory Factor Analysis (CFA) was used to check reliability and discriminant validity of collected data.

**Figure 1:**
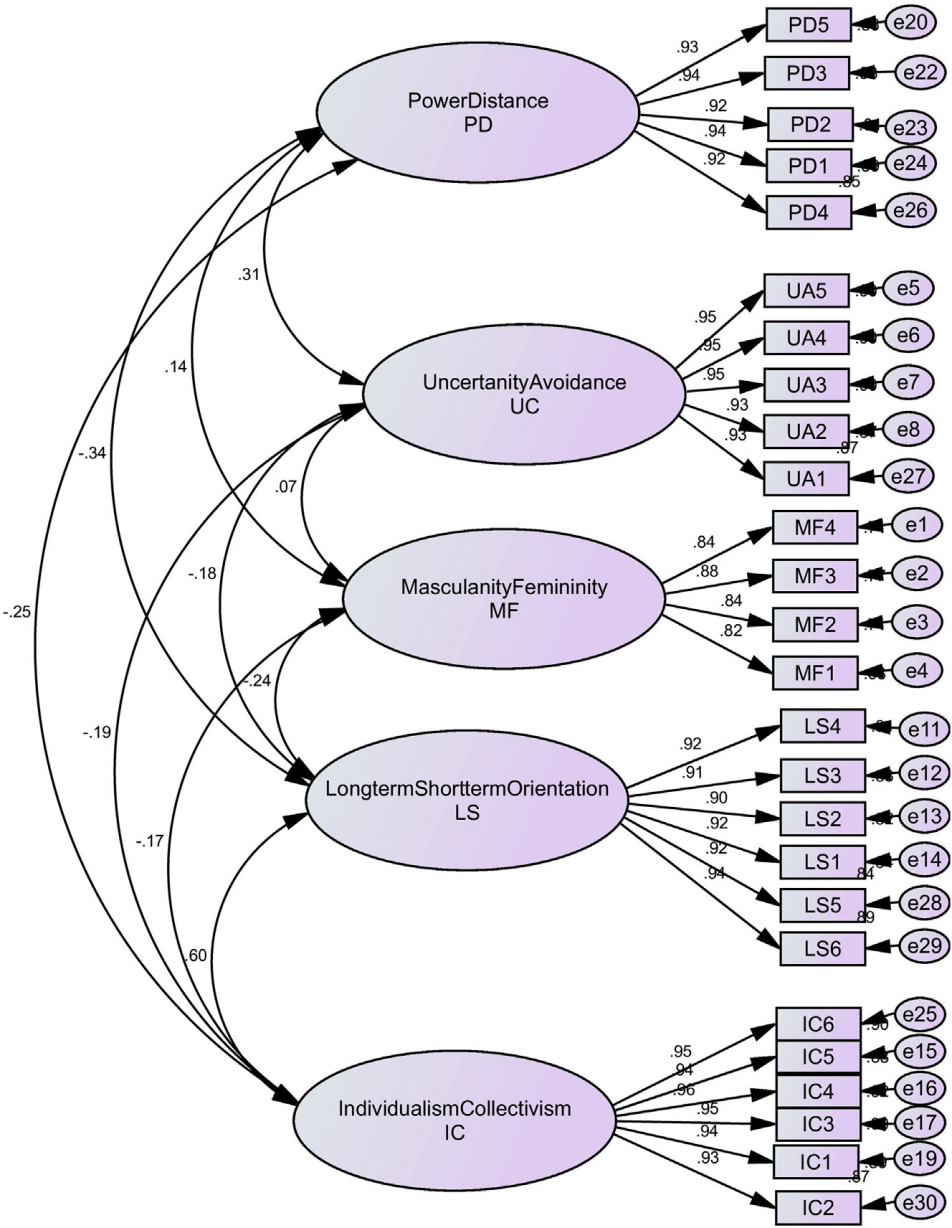
Validating our use of CV Scale Model (Yoo et al. 2011) constructs.

Kaiser-Meyer-Olkin Measure of Sampling Adequacy was used, with Varimax rotation and Maximum likelihood extraction; since AMOS was later used to test factor loading (see Figure 1). Five factors were extracted in the component matrix (see Table 1). The cumulative variance of the five factors was 84.95%, and eigenvalues for all factors were above 1; implying that extracted factors account for a large proportion of the variable's variance. The communalities for all 26 CV-Scale items were higher than 0.7, with most being higher than 0.8, suggesting that factor analysis is reliable.

**Table 1:** Factor Loading, Maximum likelihood extraction.

Items	Factor Loadings				
	1	2	3	4	5
PD1				.914	
PD2				.891	
PD3				.916	
PD4				.885	
PD5				.901	
UA1			.916		
UA2			.915		
UA3			.933		
UA4			.933		
UA5			.936		
IC1	.897				
IC2	.877				
IC3	.894				
IC4	.904				
IC5	.886				
IC6	.903				
MF1					.822
MF2					.839
MF3					.861
MF4					.829
LS1		.849			
LS2		.840			
LS3		.842			
LS4		.856			
LS5		.850			
LS6		.877			
**Cronbach's Alpha**	**.980**	**.969**	**.975**	**.969**	**.909**

Hair et al. [40] states that researchers should carefully evaluate the factor matrix (Table 1) to check that all items are loaded with values greater than 0.5. All items of our instrument loaded above 0.5, and all 26 items loaded to the relevant respective factor; in line with the research of Yoo et al. [35]. The Cronbach's Alpha of all five extracted factors was greater than 0.90 (See Table 1) demonstrating a high reliability in our CV Scale data [41]. KMO sampling adequacy values were found to be 0.935 (see Table 2) and Bartlett's test sig <0.05, which implies that the chosen variables correlated.

**Table 2:** KMO and Bartlett's test.

KMO and Bartlett's Test		
Kaiser-Meyer-Olkin Measure of Sampling Adequacy.		.935
Bartlett's Test of Sphericity	Approx. Chi-Square	17980.036
	df	325
	Sig.	.000

CFA, the second step in factor analysis, confirms the factors explored in EFA. CFA was performed using AMOS, and Figure 1 shows the measurement model of the factors of CVSCALE. Reliability in CFA is measured using composite reliability (CR), which shows the internal consistency amongst all the factors used in CFA; to measure a single construct [42]. The threshold value of CR for each single factor should be greater than 0.7. Composite reliability within our study for the five cultural factors were all above 0.9, thus confirming their reliability (see Table 3 and Figure 1).

**Table 3:** Construct reliability and validity (convergent and discriminant validity).

Constructs	CR	AVE	MSV	MF	UA	LS	PD	IC
Masculinity/Femininity (MF)	0.910	0.716	0.057	**0.846**				
Uncertainty Avoidance (UA)	0.975	0.888	0.097	0.073	**0.942**			
Long-term/Short-term Orientation (LS)	0.970	0.843	0.354	-0.239	-0.183	**0.918**		
Power Distance (PD)	0.970	0.864	0.118	0.135	0.312	-0.344	**0.930**	
Individualism/Collectivism (IC)	0.980	0.893	0.354	-0.174	-0.190	0.595	-0.245	**0.945**

Construct validity is measured using two approaches, i.e., convergent and discriminant validity [43]. Convergent validity of the construct is measured using average variance extracted (AVE). The threshold value of AVE is 0.5 [44], however the AVE, for all five factors in our study, is higher than 0.5; thus, verifying the convergent validity (see Table 3). Discriminant validity was tested, as it signifies the distinction amongst the different constructs used to measure different traits [45] The discriminant validity is established if maximum shared variance (MSV) is less than the average variance extracted (AVE) [40]. MSV of the five CV-Scale constructs is less than AVE (see Table 3).

Model fit is measured to check how well the factors in the structure correlate with the variables in the dataset. A good fit signifies that factors in the model are correct, i.e., supported by data set. Table 4 presents the model fit values obtained, and the threshold of each measure [46] Constructs of the CV-Scale were validated and confirmed. Results showed that, as in the original CV-Scale study [35] all 26-items were reliably grouped as fitting into the five factors used for further analysis. This supports the validity of our individual level data, and supports application of general results beyond Pakistan.

**Table 4:** Model fit [46].

Measures	Values	Threshold
Minimum Discrepancy per Degree of Freedom (CMIN/DF)	1.998	<3 good
Comparative Fit Index (CFI)	0.984	>0.90
Adjusted Goodness of Fit (AGFI)	0.905	>0.80
Standardised Root Mean Squared Residual (SRMR)	0.041	<0.09
Root Mean Squared Error Approximation (RMSEA)	0.044	<0.05 good, 0.05–0.10 moderate
PClose	0.973	>0.05

## 3 Results

### 3.1 Understanding the ‘average’ user

Average feedback scores, for all devices, for the eight of Kwan and Ng's Higher Education Service (HES) quality indicators, were calculated (see Table 5).

**Table 5:** Device preference of 518 student against 8 HES Quality Indicators.

Higher Education Service (HES) Quality Indicators	Face to Face	TV	Radio	Desktop/Computer	Laptop	Mobile	Tablet
Course Content	3.76	2.02	1.59	3.28	**4.03**	2.95	3.89
Facilities	3.74	1.98	1.66	3.36	**4.05**	3.06	3.20
Lecturer's Concern for Students	**4.05**	1.81	1.49	3.26	3.87	3.77	3.77
Social Activities	2.90	2.16	1.81	3.13	3.98	**4.17**	3.77
Communication with University	3.66	1.83	1.60	3.32	4.28	**4.35**	3.95
Assessment	3.53	1.59	1.43	3.22	**4.12**	3.93	3.83
Counselling Services	**4.22**	1.90	1.68	3.23	4.01	3.89	3.72
People	**4.40**	1.97	1.76	3.13	4.02	4.12	3.76

The background of the device with the highest score, for each of the eight HES indicators, is highlighted (see Table 5). Results demonstrate that students do not rate TV, Radio, Desktop, and Tablet as viable education interaction solutions. Only three delivery solutions, i.e., Laptop, Mobile, and Face-to-Face delivery were preferred. Laptop is the preferred tool for delivery of ‘Course Content’, ‘Facilities’ and ‘Assessment’; Mobile devices was preferred for organizing ‘Social Activities’ and ‘Communication with University’; and face-to-face delivery was preferred for human support indicators, i.e., ‘Lecturer's concern for students', ‘Counselling services’, and ‘People’. Results show, however, that, for 5 out of 8 services, face-to-face delivery was not identified as the student's preferred interaction solution; with the ‘average’ student instead preferring a blended solution, i.e., a combination of both traditional and face-to-face services.

### 3.2 Individual level cluster preference

Data was initially split for each demographic variable (age, gender, education level, etc.). Chi-square tests were conducted for each variable, however no significant difference was identified in learning mode or device preference as a result of general demographic factors.

The authors conducted hierarchal clustering to CV-Scale question feedback to identity whether any difference in delivery mode preference occurred as a result of variance in CV Scale cultural dimensions. To determine the number of clusters in the dataset, we tested the cubic clustering criterion and distance [47] for 1–5 cluster solutions. The cubic clustering criterion and distance increased significantly after consideration of three clusters, implying that three clusters should be used. The five validated CV-scale dimensions were used to define cluster segmentation - i.e., Power Distance (PD), Uncertainty Avoidance (UA), Individualism/Collectivism (IC), Masculinity/Femininity (MF) and Long-term/Short-term Orientation (LS). K-means clustering was applied to the full dataset, and three significant cluster segments were identified across the five CV-Scale dimensions (see Table 6).

**Table 6:** Culture at individual level cluster wise segmentation.

Cultural Dimension	Cluster Segmentation			F-Value
	Segment 1	Segment 2	Segment 3	
Power Distance (PD)	3.83	1.50	2.35	178.152∗
Uncertainty Avoidance (UA)	3.66	3.86	2.08	124.039∗
Individualism/Collectivism (IC)	1.52	2.94	4.26	376.259∗
Masculinity/Femininity (MF)	3.93	3.20	3.25	21.789∗
Long-term/Short-term Orientation (LS)	2.36	4.12	4.27	278.869∗
**Total (N)**	**146**	**175**	**197**	**518**

∗ Sign. <0.01.

The F-value for the Masculinity/Femininity dimension was low, which implies that this dimension should not be used to guide clustering. Accordingly, a student can be assigned to one of the three clusters by calculating the lowest Euclidean distance between participant and segment values (using PD, UA, IC, and LS). As a rough guide we determined clusters as:

• Cluster 1 students have the high-power distance (PD), masculinity (MF), and uncertainty avoidance (UA) scores. Literature suggests that students in cluster 1 student will most likely be assertive, controlling, self-driven, with a short-term results focus, and will expect variation in the level of service as a result of status/power/wealth held by the student.
• Cluster 2 students have the highest uncertainty avoidance (UA) score, yet the lowest PD scores. Literature suggests that students in this cluster believe more in definition of rules and structures, i.e., to avoid uncertainty. Cluster 2 would not be happy with variation in the delivery of services due to everyone being equal. Cluster 2 students are more likely to have a long-term focus when compared to cluster 1.
• Cluster 3 students have the lowest UA score, yet the highest level of collectivism - so focus on group long-term goals. Literature suggests that these students rely on the group, and work together towards the greater good of the whole. Cluster 3 participants, however, may ignore rules and fixed structures; especially if the rules or structures limit or deny implementation of the optimal solution.

The preference data for the 518 participants was sorted by cluster groups by calculating the lowest Euclidean distance for each student. Once data for each cluster was collected, the device preference data was analyzed for each cluster – see Table 1.

Cluster 1 contained 146 participants. The average cluster 1 participant preferred Face to Face delivery of material for six of the eight HES quality indicators (see Table 7); with the exceptions of ‘Communication with University’ and ‘Assessment’ which were assigned to respectively mobile and laptop.

**Table 7:** Device preference cluster 1 N = 146.

Higher Education Service (HES) Quality Indicators	Face to Face	TV	Radio	Desktop/Computer	Laptop	Mobile	Tablet
Course Content	**4.41**	2.10	1.64	3.18	3.48	2.26	3.88
Facilities	**4.45**	2.10	1.77	3.45	3.48	2.67	2.77
Lecturer's Concern for Students	**4.18**	1.91	1.49	3.19	3.22	2.93	3.62
Social Activities	**4.39**	2.21	1.82	3.11	3.70	3.54	3.75
Communication with University	3.56	1.92	1.68	3.32	4.32	**4.35**	3.86
Assessment	3.55	1.63	1.46	3.34	**4.25**	3.73	3.79
Counselling Services	**4.49**	1.98	1.71	3.18	3.78	3.57	3.66
People	**4.58**	2.18	1.87	3.20	3.96	4.10	3.74

Cluster 2 contained 175 students. The average cluster 2 participant preferred use of Mobile devices for six of the eight HES quality indicators (see Table 8). Cluster 2 participants, however, preferred Face to Face service delivery for ‘Counselling Services’ and ‘People’ HES indicators.

**Table 8:** Device preference cluster 2 N = 175.

Higher Education Service (HES) Quality Indicators	Face to Face	TV	Radio	Desktop/Computer	Laptop	Mobile	Tablet
Course Content	3.63	1.93	1.53	3.34	3.79	**4.35**	3.85
Facilities	3.38	1.91	1.57	3.29	3.95	**4.37**	2.98
Lecturer's Concern for Students	3.85	1.66	1.45	3.22	3.99	**4.46**	3.79
Social Activities	2.41	2.11	1.81	3.07	4.04	**4.75**	3.75
Communication with University	3.59	1.77	1.53	3.33	4.23	**4.34**	3.95
Assessment	3.56	1.54	1.41	3.07	3.89	**4.33**	3.83
Counselling Services	**4.13**	1.83	1.67	3.29	4.05	4.01	3.78
People	**4.27**	1.81	1.73	3.11	4.06	4.10	3.71

Cluster 3 was the largest cluster, i.e., containing 197 students. The average cluster 3 participant preferred use of Laptop for six of the eight HES quality indicators (see Table 9). Like all other groups ‘Communication with University’ and ‘People’ HES indicators were assigned respectively to Mobile and Face-to-Face delivery solutions.

**Table 9:** Device preference cluster 3 N = 197.

Higher Education Service (HES) Quality Indicators	Face to Face	TV	Radio	Desktop/Computer	Laptop	Mobile	Tablet
Course Content	3.39	2.03	1.60	3.29	**4.67**	2.22	3.93
Facilities	3.55	1.96	1.66	3.36	**4.55**	2.19	3.72
Lecturer's Concern for Students	4.14	1.85	1.52	3.35	**4.25**	3.78	3.87
Social Activities	2.24	2.16	1.80	3.20	**4.13**	4.11	3.81
Communication with University	3.78	1.81	1.60	3.32	4.30	**4.36**	4.00
Assessment	3.49	1.61	1.43	3.26	**4.23**	3.73	3.85
Counselling Services	4.11	1.90	1.66	3.22	**4.15**	4.03	3.72
People	**4.38**	1.94	1.72	3.10	4.02	4.16	3.81

## 4 Discussion and conclusions

Initial ‘average’ analysis showed that uni-directional, i.e., ‘broadcast only’ solutions, were perceived poorly by higher education students, which allows us to conclude that students no-longer see ‘receive only’ service solutions as sufficient for provision and management of student interaction - and that engagement is critical to student quality perception. During the Covid-19 global pandemic many institutions were forced to shift their teaching practices online. Although most HEIs were able to facilitate online uni-directional dissemination of teaching material, our research shows that additional focus needs to be place on encouraging level of student interaction, and/or personalization of, e-learning provision.

When demographic variables were analyzed, no significant differences were identified in learning mode or device preference as a result of demographic variables (age, gender, education level groups), however when split into clusters, using CV scale dimensions (see Table 6), considerable differences were identified (see Tables 7, 8, and 9). By considering the CV (Cultural Value) Scale dimensions, our results highlighted three distinct technology preference clusters: i) students, with a high-power distance score, who prefer traditional face-to-face teaching methods; ii) students with low power distance and high uncertainty avoidance scores who prefer use e-learning on their mobile devices, and iii) students with low power distance and low uncertainty avoidance scored who prefer use laptop devices. The significant difference between cluster preference shows that a one-size-fits-all approach to e-learning service provision doesn't align to individual information assimilation needs.

Although the average student is happy with, and actually prefers, the use of a blended approach in the provision of education services (see Table 1), however the concept of the ‘average’ student is non-sensical. HEIs need to ensure that they implement solutions that are capable of considering individual differences and needs; or they risk student rejection, loss of reputation, and loss of revenue. Student quality perception is linked to satisfaction, and satisfaction is linked to engagement. As such educators need to continue to investigate what factors drive student satisfaction, and how these factors can be actively enhanced in used e-learning solutions. E-learning solution should not be used by HEIs as a quick low-cost solution for the masses, but as part of an interactive, personalized, and blended educational experience.

## 5 Limitations and future research

Data collection in our study was limited to university students studying Bachelors and Masters Programs. To gain a more comprehensive understanding of preference variation, data collection needs to be extended to other levels of education and countries. Consideration at other levels of education would help the researchers to identify when a difference in device preference first occurs. Such knowledge would help early years educators to either i) support personalized interaction preference, or ii) support skills development to maximize non-preference engagement. Similarly, the collecting of data from multiple countries would help in the international validation of learning clusters and/or support in the development of best practice guidelines.

Although there is still much work to be done, i.e. to appreciate how student segmentation can be practically used, and/or how personalization interaction models can be developed, this study has clearly highlighted that e-learning system developers, academic faculty staff, policy makers, and administrative staff, all need to appreciate that interaction is essential to developing a positive student quality perception, and satisfying individual differences is critical to delivering e-learning services that maximize student retention and satisfaction.

## Declarations

### Author contribution statement

Samnan Ali: Conceived and designed the experiments; Performed the experiments; Analyzed and interpreted the data; Contributed reagents, materials, analysis tools or data; Wrote the paper.

Stephen Gulliver: Conceived and designed the experiments; Wrote the paper.

Muhammad Basir: Analyzed and interpreted the data; Contributed reagents, materials, analysis tools or data.

Amaad Uppal: Contributed reagents, materials, analysis tools or data.

### Funding statement

This research did not receive any specific grant from funding agencies in the public, commercial, or not-for-profit sectors.

### Data availability statement

Data will be made available on request.

### Declaration of interests statement

The authors declare no conflict of interest.

### Additional information

No additional information is available for this paper.

## Appendix A Supplementary data

TECH_Preference_Appendix TECH_Preference_Appendix
