# Supplementary material for: Research investigating individual device preference and e-learning quality perception: can a one-solution-fits-all e-learning solution work?
Source: Heliyon. 2021 Jun 18;7(6):e07343. doi: 10.1016/j.heliyon.2021.e07343 (PMC8239717; doi:10.1016/j.heliyon.2021.e07343)
Supplement: TECH_Preference_Appendix [file mmc1.docx]

Appendix A

Matrix used, within questionnaire part 2, to capture feedback concerning student perception concerning learning mode and e-learning devices.

| **Indicators** | **Face to Face** | **TV** | **Radio** | **Desktop / Computer** | **Laptop** | **Mobile** | **Tablet** |
| --- | --- | --- | --- | --- | --- | --- | --- |
| 1. Course content |  |  |  |  |  |  |  |
| 1. Facilities |  |  |  |  |  |  |  |
| 1. Lecturer’s Concern for Students |  |  |  |  |  |  |  |
| 1. Social activities |  |  |  |  |  |  |  |
| 1. Communication with University |  |  |  |  |  |  |  |
| 1. Assessment |  |  |  |  |  |  |  |
| 1. Counselling Services |  |  |  |  |  |  |  |
| 1. People |  |  |  |  |  |  |  |

Appendix B

Strongly Disagree (SD), Disagree (DA), Neutral (N), Agree (A), Strongly Agree (SA)

| **Cultural Value Scale** | **SD** | **DA** | **N** | **A** | **SA** |
| --- | --- | --- | --- | --- | --- |
| **Power Distance** |  |  |  |  |  |
| 1. People in higher positions should make most decisions without consulting people in lower positions. |  |  |  |  |  |
| 1. People in higher positions should not ask the opinions of people in lower positions too frequently. |  |  |  |  |  |
| 1. People in higher positions should avoid social interaction with people in lower positions. |  |  |  |  |  |
| 1. People in lower positions should not disagree with decisions by people in higher positions. |  |  |  |  |  |
| 1. People in higher positions should not delegate important tasks to people in lower positions. |  |  |  |  |  |
| **Uncertainty Avoidance** |  |  |  |  |  |
| 1. It is important to have instructions spelled out in detail so that I always know what I'm expected to do. |  |  |  |  |  |
| 1. It is important to closely follow instructions and procedures. |  |  |  |  |  |
| 1. Rules and regulations are important because they inform me of what is expected of me. |  |  |  |  |  |
| 1. Standardized work procedures are helpful. |  |  |  |  |  |
| 1. Instructions for operations are important. |  |  |  |  |  |
| **Individualism/Collectivism** |  |  |  |  |  |
| 1. Individuals should sacrifice self-interest for the group (either at school or the work place). |  |  |  |  |  |
| 1. Individuals should stick with the group even through difficulties. |  |  |  |  |  |
| 1. Group welfare is more important than individual rewards. |  |  |  |  |  |
| 1. Group success is more important than individual success. |  |  |  |  |  |
| 1. Individuals should only pursue their goals after considering the welfare of the group. |  |  |  |  |  |
| 1. Group loyalty should be encouraged even if individual goals suffer. |  |  |  |  |  |
| **Masculinity/Femininity** |  |  |  |  |  |
| 1. It is more important for men to have a professional career than it is for women. |  |  |  |  |  |
| 1. Men usually solve problems with logical analysis; women usually solve problems with intuition. |  |  |  |  |  |
| 1. Solving difficult problems usually requires an active, forcible approach, which is typical of men. |  |  |  |  |  |
| 1. There are some jobs that a man can always do better than a woman. |  |  |  |  |  |
| **Long-term Orientation/Short-term Orientation** |  |  |  |  |  |
| 1. Careful management of money (Thrift). |  |  |  |  |  |
| 1. Going on resolutely in spite of opposition (Persistence). |  |  |  |  |  |
| 1. Personal steadiness and stability. |  |  |  |  |  |
| 1. Long-term planning. |  |  |  |  |  |
| 1. Giving up today's fun for success in the future. |  |  |  |  |  |
| 1. Working hard for success in the future. |  |  |  |  |  |
